# Evaluation of Physically and/or Chemically Modified Chitosan Hydrogels for Proficient Release of Insoluble Nystatin in Simulated Fluids

**DOI:** 10.3390/gels8080495

**Published:** 2022-08-10

**Authors:** Andra-Cristina Enache, Corneliu Cojocaru, Petrisor Samoila, Adrian Bele, Andra-Cristina Bostanaru, Mihai Mares, Valeria Harabagiu

**Affiliations:** 1 “Petru Poni” Institute of Macromolecular Chemistry, 41A Grigore Ghica Voda Alley, 700487 Iasi, Romania; 2 Laboratory of Antimicrobial Chemotherapy, “Ion Ionescu de la Brad” University, 8 Mihail Sadoveanu Alley, 700489 Iasi, Romania

**Keywords:** hydrogels, chemical cross-linking, chitosan, micronized nystatin, antifungal activity

## Abstract

To avoid fungal spreading in the bloodstream and internal organs, many research efforts concentrate on finding appropriate candidiasis treatment from the initial stage. This paper proposes chitosan-based physically or chemically cross-linked hydrogels aimed to provide sustained release of micronized nystatin (NYSm) antifungal drug, known for its large activity spectrum. Nystatin was demonstrated itself to provide hydrodynamic/mechanic stability to the chitosan hydrogel through hydrophobic interactions and H-bonds. For chemical cross-linking of the succinylated chitosan, a non-toxic diepoxy-functionalized siloxane compound was used. The chemical structure and composition of the hydrogels, also their morphology, were evidenced by infrared spectroscopy (FTIR), by energy dispersive X-ray (EDX) analysis and by scanning electron microscopy (SEM), respectively. The hydrogels presented mechanical properties which mimic those of the soft tissues (elastic moduli < 1 MPa), necessary to ensure matrix accommodation and bioadhesion. Maximum swelling capacities were reached by the hydrogels with higher succinic anhydride content at both pH 7.4 (429%) and pH 4.2 (471%), while higher amounts of nystatin released in the simulative immersion media (57% in acidic pH and 51% in pH 7.4) occurred from the physical cross-linked hydrogel. The release mechanism by non-swellable matrix diffusion and the susceptibility of three *Candida* strains make all the hydrogel formulations effective for NYSm local delivery and for combating fungal infections.

## 1. Introduction

The prevalence of fungal invasive infections is continuously increasing as a result of the growing number of immunocompromised patients, having an estimated attributable global mortality higher than 1.5 million deaths every year [1,2,3]. Superficial mycotic infections caused by *Candida* species often affect the mucocutaneous tissues (oral or vulvovaginal mucosa) [4,5]. This is why the challenge of the last decades was concerned with designing new therapeutic systems for candidiasis treatment from the initial stage, in order to prevent fungal spreading in the bloodstream and internal organs [6,7]. 

Nystatin is known for a wide range of antifungal activity, being intended for the prophylaxis and treatment of candidiasis located on the skin and mucous membranes [8]. Derived from *Streptomyces noursei* actinobacterium, the polyene macrolide possesses a macrolactone ring structure with a polyketide core [9]. Acting like an ionophore, nystatin is highly selective towards fungi, interacting with the membranes’ ergosterol and forming pores that allow K+ ions to pass through, resulting in the death of fungal cells [10]. However, the antifungal drug presents a series of drawbacks that limit their usage, such as low solubility and the host-induced toxicity they come with [11]. To overcome these disadvantages, micronized nystatin (NYSm) with particle dimensions up to 10 microns was manufactured to facilitate its use in suspensions [12]. Additionally, a challenge consists in developing an appropriate therapeutic system for the antifungal drug delivery, as it must be biocompatible, available, easy to administer, adherent to mucocutaneous tissue and able to retain the drug for prolonged release.

The uniqueness of hydrogels in terms of their physical properties makes them ideal candidates for embedding, carrying and releasing drugs and other biologically active substances [13]. The ability to absorb large amounts of water and their flexibility are properties that make hydrogels closely resemble biological tissues [14]. Moreover, another advantage is given by the versatility of preparation methods, as the hydrogels can be obtained either by physical cross-linking (based on the intermolecular hydrophobic interactions, electrostatic attraction or hydrogen bonding), or by permanent chemical cross-linking of the polymer chains through covalent bonding between functional groups [15,16]. As has been shown by others, hydrogel formulations are ideal drug delivery systems for topical application in the treatment of fungal infections [17,18,19].

Properties such as biocompatibility, bioactivity, biodegradability, low costs and the ease of processing under different formulations due to the presence of different reactive functional groups (hydroxyl, amino and carboxyl) able to undergo physical and chemical cross-linking, also the unique chemical structure, recommend polysaccharides for their use in hydrogel-based therapeutic formulations [20]. Among these, chitosan (CS) cationic polysaccharide arouses interest in development of pharmaceutical hydrogels for mucocutaneous (buccal, vaginal) drug delivery [21]. Obtained by alkaline or enzymatic degradation of chitin, the polycation structure consists of d-glucosamine units (deacetylated) and *N*-acetyl-d-glucosamine units (acetylated) connected by β-1,4-glycosidic bonds [22]. The abundant presence of functional groups (–NH_2_ at C2 and –OH at C3 and C6) provides both chemical reactivity and bioactivity to the chitosan chains [23]. As for the latter, chitosan is well-known for possessing mucoadhesive properties, due to the strong electrostatic interaction between positively charged amino groups and negatively charged epithelial surfaces and sialic acid (component of mucus) [24].

Chitosan-based antifungal formulations containing propolis and/or standard nystatin were investigated in a previous work [25], and the efficiency of chitosan-nystatin hydrogel stabilization by physical interactions was observed. In this context, this study aims first to deepen the types of physical interactions between chitosan and nystatin that underlie the formation of the hydrogel. Secondly, the development of new approaches is pursued, based on the chemical modification of the polysaccharide by combining succinylation (with succinic anhydride—SA) and cross-linking (with an epoxy-terminated disiloxane—DS) in order to provide new properties to the final formulations for antifungal activity evaluation. 

## 2. Results and Discussion

### 2.1. Molecular Docking Simulation of Physical Interactions between Chitosan and Nystatin

**Figure 1:**
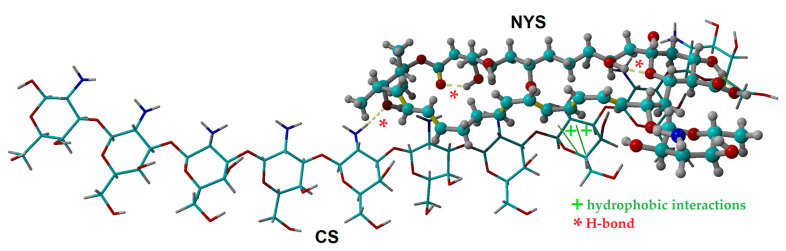
Molecular docking between chitosan (CS) and nystatin (NYS): best pose of the docked complex highlighting the binding mode between CS oligomer (receptor) and NYS molecule (ligand).

In order to detail some insights regarding the molecular interaction mechanism between the chitosan macromolecule (CS) and nystatin (NYS), the molecular docking simulation technique was employed. In general, molecular docking represents a computing simulation technique for predicting the binding mode and orientation of a ligand (e.g., organic molecule) when it interacts with a receptor (e.g., macromolecule, oligomer or supramolecular aggregate). In our case, a chitosan oligomeric chain (made of ten monomeric units) was modeled as the receptor, while a nystatin (NYS) molecule was modeled as the ligand. Outcomes of the molecular docking calculations are highlighted in Figure 1, where the best docking pose between CS (receptor) and NYS (ligand) is detailed. For this best pose of the docked complex (CS-NYS), the energy score (affinity) was found to be −8.24 kcal/mol and the dissociation constant was equal to 0.905 μM. Moreover, the interaction energies between the CS receptor and NYS ligand were estimated at the level of the YASARA force field [26]. Hence, the computational results suggested that the physical interaction was predominant, because of low Van-der-Waals interaction energy (−8.17 kcal/mol) compared to the high electrostatic (Coulumb) interaction energy (+7.51 kcal/mol). Moreover, the molecular docking simulations suggested that the docked complex (CS-NYS) was stabilized by hydrophobic (>CH…HC<) interactions and the intermolecular hydrogen-bond (H-bond) formation between the receptor (CS) and ligand (NYS), as shown in Figure 1. The formation of the intramolecular H-bonds in the structure of the ligand (NYS) was also evidenced by the molecular docking simulation. 

### 2.2. Chemical and Physical Hydrogels by Chitosan Modification

As demonstrated in a previous work [25] the dissolution of chitosan in lactic acid is providing satisfactory mechanical characteristics adequate for the local application of the resulted formulations onto soft mucocutaneous tissues. Micronized nystatin was dispersed in glycerin using a mortar and a pestle [27], thus facilitating the in situ introduction of the drug in the chitosan-lactic acid (CS-LA) matrix and stabilizing the hydrogel structure by inter/intramolecular hydrogen bonds (mainly between H atoms from –NH_2_ groups of the polysaccharide and lone electron pair O atoms from nystatin) and hydrophobic interactions, as observed in Figure 1. Moreover, glycerin was used to increase the flexibility and toughness of the chitosan dried hydrogels, also demonstrated by others [25,28].

**Figure 2:**
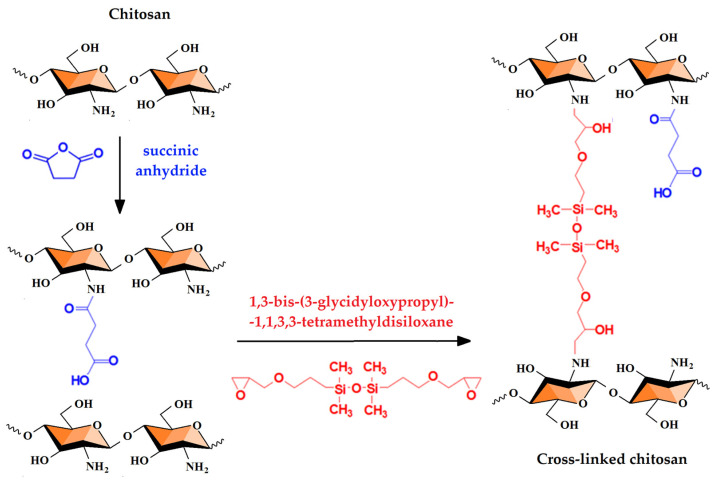
Schematic representation of chitosan chemical modification by succinylation with succinic anhydride (SA) and cross-linking with epoxy-terminated disiloxane (DS).

As shown in Figure 2, preparation of chemical hydrogels involved in the first stage include the partial succinylation of CS with succinic anhydride (SA), as it reacts easily with the primary –NH_2_ groups without producing side reactions and also leads to a non-toxic modified polymer, but with increased solubility at neutral pH [29]. In the second stage, cross-linking with 1,3-bis-(3-glycidyloxypropyl)-1,1,3,3-tetramethyldisiloxane (DS) is pursued by covalent bond formation between some of the free amino groups and epoxy groups of the DS. The latter aims to provide an amphiphilic character to the final hydrogel by the introduction of hydrophobic disiloxane units, and to increase its mechanical and hydrodynamic stability. At the same time, DS offers the possibility to adjust the tissue adhesion of the film and the hydrophobic/hydrophilic ratio, depending on the content of the cross-linking agent. Moreover, the amphiphilic nature of final formulations facilitates the stabilization and transport of drugs that contain polar and non-polar sequences in the structure, such as nystatin [30]. The total percentage of succinylated and cross-linked amino groups of CS (84%) was calculated in order to ensure the presence of free –NH_2_ groups, which are needed for the interaction with the negatively charged functional groups of mucus (mucoadhesivity).

The final films’ formulations (Table 1) obtained by different CS cross-linking methods (physical and chemical) were comparatively characterized regarding the mechanical properties, swelling behavior and in vitro NYSm release in two solutions simulating the oral and vaginal fluids (with pH 7.4 and pH 4.2, respectively) [31] with the purpose of evaluating their capability of delivering the antifungal drug by the buccal/vaginal route of administration. The antifungal activity of all the NYSm-loaded hydrogels was also investigated. 

### 2.3. Structural Characterization by FTIR Spectroscopy

**Figure 3:**
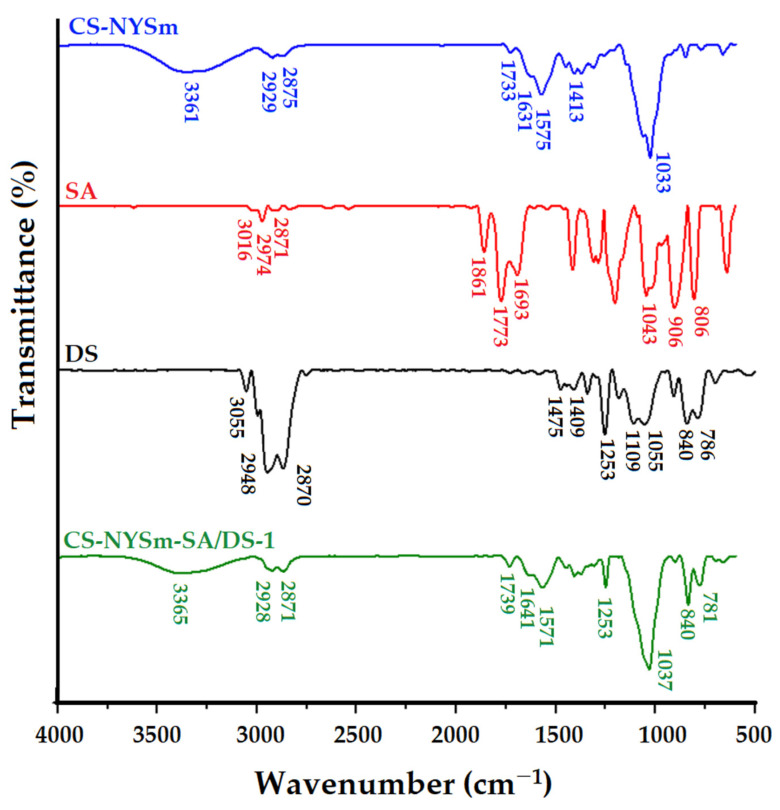
FTIR spectra of micronized-nystatin-charged chitosan film (CS-NYSm), succinic anhydride (SA), epoxy-terminated disiloxane cross-linking agent DS and chemically modified NYSm-chitosan film (CS-NYSm-SA/DS-1).

Structural properties of the raw materials, also of the prepared hydrogels, were investigated by FTIR spectroscopy. CS-LA, NYSm and CS-NYSm spectra are presented in Figure S1 in Supplementary Materials (SM), where they are briefly discussed, being analyzed in-depth in a previous work where we obtained similar results for formulations prepared by a different method [25]. Figure 3 compares the FTIR spectra of the CS-NYSm and CS-NYSm-SA/DS-1 formulations (similar FTIR spectra were also obtained for CS-NYSm-SA/DS-2). The spectra of raw materials (SA and DS) are also added for easier understanding of the IR adsorptions of the chemically cross-linked formulation.

In the spectrum of succinic anhydride, the main absorption bands attributed to the asymmetric and symmetrical C–H stretching vibrations (2974 and 2871 cm^−1^) and of the C–H vibrations specific to the cyclic anhydride (3016 cm^−1^) can be observed. The absorptions bands from 1863 cm^−1^ and 1773 cm^−1^ correspond to the symmetrical and asymmetrical stretching vibrations of the C=O group, respectively, characteristic for the anhydrides. The band from 1689 cm^−1^ is given by the symmetrical stretching vibrations of the C=O group of the partially hydrolyzed succinic anhydride. The absorption bands from 1043 cm^−1^ and 906 cm^−1^ are specific to C–O–C vibrations for anhydride with five carbon atoms in the ring [32]. The DS spectrum also reveals characteristic absorption bands given by C–H stretching vibrations specific to the epoxy group at 3055 cm^−1^, Si–CH_3_ stretching vibrations (1253 cm^−1^ and the doublet 840–786 cm^−1^) and Si–O–Si vibrations in the region of 1053–1109 cm^−1^, superimposed over the C–O–C bands in the glycidyloxypropyl unit [33].

The successful succinylation of CS is demonstrated by the disappearance of the absorption bands from 3016 cm^−1^, from 1863 cm^−1^ and from 1773 cm^−1^ in the spectrum of CS-NYSm-SA/DS-1 as a result of the anhydride ring opening [34,35]. The cross-linking process with DS was confirmed by the presence of absorption bands from 1253, 840 and 781 cm^−1^, characteristic of the siloxane sequence, while the Si–O–Si band is superposed on the C–O–C groups’ absorption bands coming from CS, SA and NYSm.

### 2.4. Morphological Evaluation of CS Films by SEM and EDX

**Figure 4:**
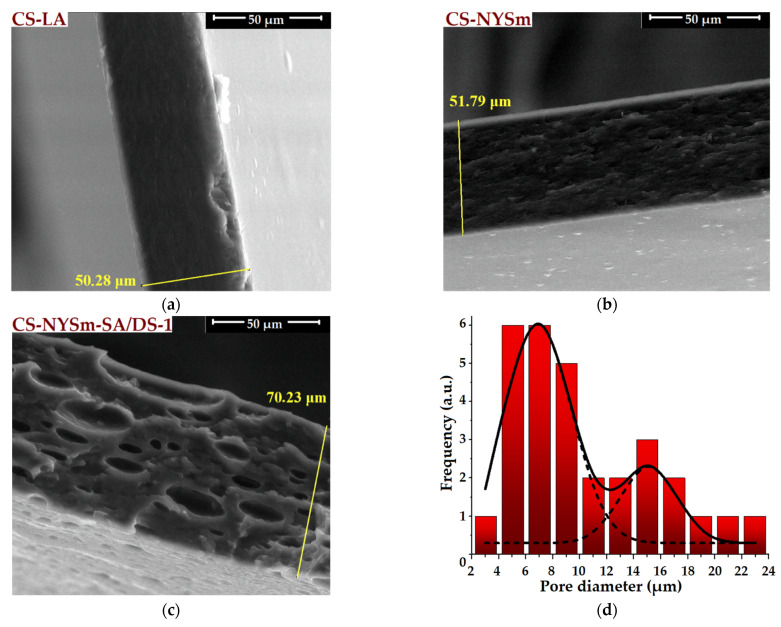
Cross-section morphologies of: (**a**) unmodified chitosan film (CS-LA); (**b**) nystatin-loaded chitosan film (CS-NYSm), (**c**) DS cross-linked film (CS-NYSm-SA/DS-1) and (**d**) the histogram of pore distribution corresponding to CS-NYSm-SA/DS-1 film (fitted with Gaussian function in OriginPro 8.5).

Micronized nystatin powder was morphologically examined using scanning electron microscopy (SEM). Figure S2a (SM) reveals the NYSm granular structure with a tendency of forming aggregates, while the particle size distribution histogram (Figure S2b in SM) confirms the micron-sized dimensions of the NYSm crystallites (between 0.5 and 5 µm). SEM microscopy was also used to evaluate the morphology of NYSm-loaded chitosan films by reference to CS-LA film (see the cross-section (Figure 4) and surface (Figure S3 in SM) SEM images). As shown in Figure 4a, CS-LA film presents a specific homogenous cross-section morphology [25,36], while the addition of micronized nystatin to the polymer matrix (CS-NYSm) led to a rougher cross-section morphology, with the occurrence of fine folds (Figure 4b). However, the uniform dispersion of NYSm crystallites into the CS matrix and successful embedment of the antifungal drug is easily observed.

By reference to the microscopic cross-section of CS-LA and CS-NYSm films, it can be noticed that the introduction of succinic anhydride (SA) and disiloxane cross-linking agent (DS) into the polysaccharide matrix (CS-NYSm-SA/DS-1) generates the appearance of unevenly dimensional pores distributed over the entire thickness of the section (Figure 4c). This morphology is explained by phase separation induced by the presence of hydrophobic disiloxane sequences in the polymer matrix, given the polar character of the separation medium (water and lactic acid solution) [33]. In order to evaluate the pore diameters for the CS-NYSm-SA/DS-1 film, the pore size distribution histogram was given in Figure 4d. Thus, the cross-linked chitosan film is characterized by pores with a diameter between 2 and 24 µm and presented a bimodal Gaussian pore distribution, as a result of the phase separation induced by the disiloxane component. This type of distribution is also encountered in the literature [37]. Moreover, according to the cross-section SEM images given in Figure 4a–c, an increasing trend in the thicknesses of the obtained films was noticed. Compared to the CS-LA film (50 µm), the thickness of the CS-NYSm films slightly increased with the drug embedment, while the cross-linking of chitosan with DS led to an increase in the film thickness of about 20 µm (CS-NYSm-SA/DS-1). Similar effects were observed by other authors [38] on superporous materials obtained by complexation from *N*,*O*-carboxymethyl chitosan and polydimethylsiloxane functionalized with hydroxyalkyl groups.

The surface morphology of the obtained films (Figure S3 in SM) is in accordance with their cross-section structure. Thus, the homogeneity and uniformity typical for unmodified chitosan film (Figure S3a) was observed, given by the solvent evaporation drying method, the non-porous surface of CS-NYSm film (Figure S3b) and the separation in microphases on the CS-NYSm-SA/DS-1 surface (Figure S3c), due to the incompatibility between the DS hydrophobic component and the CS hydrophilic one. Additionally, the cross-linked film showed a large number of well-defined pores of the order of microns on the surface, ranging from 1 to 30 µm due to the coalescence of hydrophobic molecules (Figure S3c).

The composition of the hydrogels was qualitatively evaluated by EDX (Figure S4). The presence of Si atoms was noticed, besides the specific C, O and N atoms of the chitosan and NYSm-glycerin component, confirming the presence of the cross-linking DS agent in the CS-NYSm-SA/DS-1 formulation (Figure S4c). Similar morphologies were also identified for CS-NYSm-SA/DS-2. 

### 2.5. Mechanical Properties of Dried Hy Drogels

The mechanical characteristics of the chitosan films were assessed in order to demonstrate their proper applicability in soft tissue use, as required by therapeutic applications for the treatment of oral and/or vaginal candidiasis. The characteristic stress-strain curves are shown in Figure S5, while the tensile strength at break, elongation at break and modulus of elasticity (Young’s modulus) values are expressed in Table 2. 

As compared to the CS-LA film, all the prepared nystatin-containing chitosan films showed ductile properties, with a major decrease in the tensile strength and modulus of elasticity (from 101 MPa to 1–3 MPa and from 43 MPa to 0.1–0.4 MPa, respectively). The highest value of elongation at break was observed for the physically cross-linked CS-NYSm formulation. All the investigated NYSm-charged films presented an increased elasticity, mainly due to the glycerin content [25,28]. 

The presence of succinic anhydride and a cross-linking agent led to a significant decrease in tensile strength and elongation at break, being an effect of the chemical modification of chitosan chains by formation of covalent bonds, much stronger than the non-covalent interactions that stabilize the CS-NYSm. These results can also be correlated with the presence of the porous structure of the chitosan films modified with SA and DS, as was observed in SEM images from Figure 4c and Figure S3c. Moreover, it can be noticed that films with a higher content of cross-linking agent presented an increased elasticity at break and low modulus of elasticity compared to films with low amounts of DS. This trend is also found in the literature, knowing that polysiloxanes induce an increase in flexibility to chitosan films [39]. All the dried antifungal formulations showed improved mechanical properties in terms of increased elasticity and flexibility (as compared with pristine chitosan film), which is necessary to ensure the CS matrix accommodation on the physiological tissue, and, most importantly, to improve the antifungal film’s adherence on the site of action. Moreover, the Young modulus values for the NYSm-loaded chitosan films are similar to those of soft biological tissues (less than 1 MPa), which indicates a biomimetic character, making the dried hydrogels suitable for their application on the buccal/vaginal mucosa and drug delivery [40].

### 2.6. Swelling Behavior of NYSm-Loaded Dried Hydrogels

**Figure 5:**
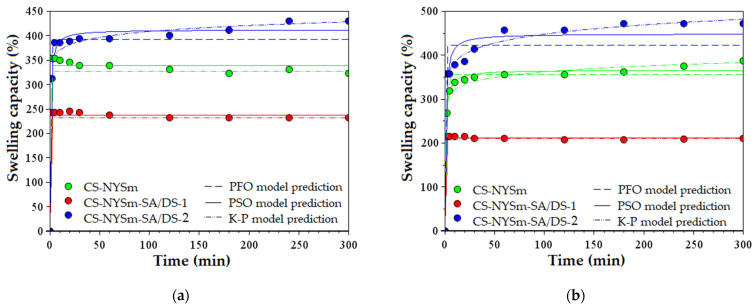
Swelling capacity of micronized nystatin-containing chitosan films (CS-NYSm, CS-NYSm-SA/DS-1 and CS-NYSm-SA/DS-2) evaluated in PBS solution of: (**a**) pH 7.4, (**b**) pH 4.2.

The swelling capacity of nystatin-charged chitosan films was evaluated under simulated conditions at 37 °C by immersion in PBS solutions corresponding to biological fluids, such as saliva (pH 7.4) and vaginal fluid (pH 4.2). The swelling kinetics are graphically represented in Figure 5a and Figure 5b, respectively. It should be mentioned that the CS-LA film has entirely dissolved into the aqueous solution, while the nystatin-loaded formulations were able to maintain their hydrogel structure due to the physical and/or chemical cross-linking of the polymer chains, even after 24 h of immersion. 

As expected and shown in Figure 5, maximum swelling capacities were reached by the CS-NYSm-SA/DS-2 film in both pH 7.4 (429%) and pH 4.2 (471%), due to the higher succinic anhydride content and lower cross-linking degree. As described in the literature, succinylation of chitosan induces an increase in its solubility at pH values lower than 4.5 and higher than 7, due to the protonation of the amino groups in the acidic medium and to the formation of carboxylate ions (–COO^−^) in neutral to basic medium, respectively [41]. In contrast, the introduction of a larger amount of cross-linker into the polysaccharide matrix resulted in a decrease of the swelling capacity of CS-NYSm-SA/DS-1 films to 231% (pH 7.4) and 200% (pH 4.2). This correlation between swelling capacity and SA/DS content is in accordance with the literature, knowing that chitosan succinylation increases hydrogels hydrophilicity [42], and increasing the amount of hydrophobic disiloxane cross-linking agent inhibits the CS film swelling in PBS solution [43]. Thus, the CS-NYSm-SA/DS-1 film had lower values of maximum absorption capacity (323% at pH 7.4 and 387% at pH 4.2).

Regarding the CS-NYSm film obtained only by the physical interactions, it can be noticed that the maximum swelling capacities exceed the values of the strongly cross-linked CS-NYSm-SA/DS-1 film. In addition, the swelling behavior for all the NYSm-charged films exhibited an instant increase in the first minutes, very close to their maximum swelling capacity.

The parameters of the kinetic pseudo-first order (PFO), pseudo-second order (PSO) and Korsmeyer–Peppas (K–P) models are reported in Table S1 (SM) for all the NYSm-containing chitosan films, at both values of pH. The theoretical values of swelling capacity at equilibrium (S_e1_ and S_e2_) are in accordance with the experimental values obtained after 5 h of the films’ immersion in PBS solutions. Moreover, the K–P diffusion exponent (n) indicates Fickian diffusion-controlled swelling kinetics (n values much lower than 0.5), as water molecules diffuse in the polymeric matrix faster than the relaxation of the CS chains occurs [44,45,46].

### 2.7. Micronized Nystatin In Vitro Release from the Antifungal Film Formulations

**Figure 6:**
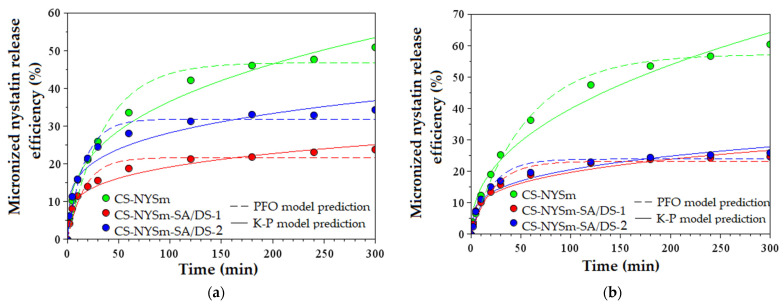
In vitro micronized nystatin release from hydrogel films (CS-NYSm, CS-NYSm-SA/DS-1 and CS-NYSm-SA/DS-2) evaluated in PBS solution of: (**a**) pH 7.4 and (**b**) pH 4.2.

In order to simulate the pH of the two fluids provided as modalities for the administration of nystatin in the treatment of oral or vaginal candidiasis (saliva and vaginal fluid), the in vitro release of micronized nystatin was evaluated in PBS solutions at pH 7.4 (Figure 6a) and pH 4.2 (Figure 6b), respectively. From a clinical point of view, an initial rapid release of nystatin in the early stage is beneficial, as it helps reach a therapeutic concentration of the drug in the shortest time, and the subsequent sustained release helps to maintain a minimum effective concentration. For this reason, the nystatin-release kinetics were measured and graphically represented for a period up to 5 h (as seen in Figure 6). The release kinetics of nystatin are characterized by a “burst effect”, which implies a quick nystatin release in the first 30 min (about half of the quantity released during 5 h), followed by a slower release up to 5 h, most pronounced for the chemically cross-linked hydrogels. CS-NYSm showed the best prolonged effect of nystatin release in both neutral and acidic pH, while chemically cross-linked samples indicated a prolonged effect only in neutral pH. The nystatin release efficiency was also measured after 24 h to determine whether a minimum effective concentration was maintained in that time (Table S2). It was observed that the antifungal drug release continues to rise after 5 h, but only by 1–6% in neutral pH and by 9–15% in acidic medium. Comparing the methods of cross-linking the CS chains, one may see that the non-covalent interactions (hydrogen bonds, hydrophobic and electrostatic interaction) lead to faster and higher amounts of NYSm release (from CS-NYSm film) in both immersion media (57% in acidic pH and 51% in pH 7.4), while the succinylated and cross-linked chitosan matrix presents lower release capacities. Moreover, the CS-NYSm film presented increased release capacity at pH 4.2, due to the presence of free amino groups of CS and their protonation in an acidic medium. As expected, increasing the cross-linker amount in the CS-NYSm-SA/DS-1 film, by comparison with CS-NYSm-SA/DS-2 film, results in decreasing NYSm release efficiency (from 33% to 23% at pH 7.4 and from 25% to 24% at pH 4.2). 

The parameters of the PFO and K–P kinetic models are presented in Table S2. PFO fitting indicates that the experimental data are in accordance with the theoretical ones, providing a good estimation of the nystatin release efficiency (%) [45]. Moreover, the K–P kinetic release model suggests that release the mechanism occurs by non-swellable matrix diffusion [46,47]. According to the *n* parameter values ranging from 0.23 to 0.43 (n < 0.5), it can be mentioned that the transport mechanism of NYSm from the hydrogels is based on quasi-Fickian diffusion [47,48].

### 2.8. Antifungal Activity of the Hydrogels against Candida spp.

**Figure 7:**
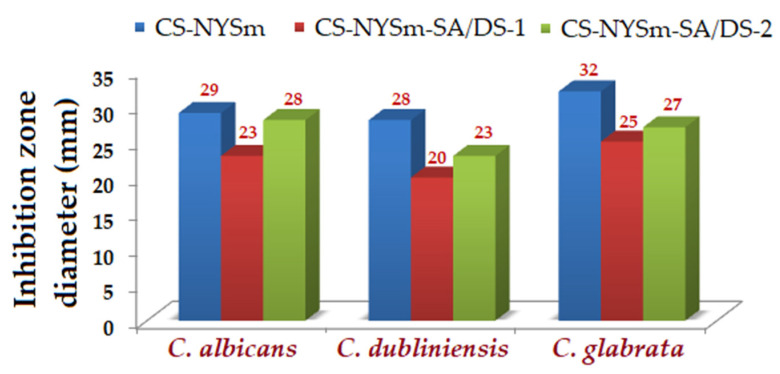
Inhibition zones diameters (mm) from the antifungal activity evaluation of CS-NYSm, CS-NYSm-SA/DS-1 and CS-NYSm-SA/DS-2 hydrogels against *Candida albicans*, *Candida dubliniensis* and *Candida glabrata*, respectively.

Micronized-nystatin-loaded hydrogels showed a pronounced antifungal effect against *Candida albicans*, *Candida dubliniensis* and *Candida glabrata* strains, with clear areas of inhibition measuring between 20 and 32 mm (Figure 7). The antifungal activity of NYSm-charged hydrogels against the three *Candida* strains was evaluated by using the agar disk diffusion method, as shown in Figure S6. All the tested CS hydrogels exhibited an antifungal effect consistent with the in vitro release of nystatin results. Thus, physically cross-linked chitosan hydrogel CS-NYSm showed increased inhibitory activity, mainly due to the presence of positively charged amino groups of CS and their interactions with the negatively charged fungal membrane. This mechanism of action by electrostatic attraction is known to induce damage to cell membranes [24]. However, chemically cross-linked CS hydrogels manifested antifungal activity close to those of physical gels, especially in the case of the CS-NYSm-SA/DS-2 hydrogel. This might be explained by the higher amount of succinic anhydride content, which can additionally lead to the H bond formation between the carboxylic groups of the succinylated chitosan and the –OH group of the fungal membrane sterols, causing pore formation and cellular leakage [49]. 

## 3. Conclusions

Two different hydrogel formulations were obtained. The mechanical/hydrodynamic stability of the first system is provided by hydrophobic (>CH…HC<) interactions and intermolecular hydrogen-bond (H-bond) formations between CS chains and the NYSm macrocycle. CS-NYSm-SA/DS-1 and CS-NYSm-SA/DS-2 hydrogels were obtained by CS chemical modification by combining succinylation with SA and cross-linking with DS epoxy functional units. All the prepared antifungal hydrogel formulations have shown increased elasticity and flexibility, which mimic those of the soft tissues (Young’s modulus less than 1 MPa), making the antifungal systems suitable for their local application in mucocutaneous candidiasis. A pronounced “burst-effect” release followed by a sustained release up to 24 h was found for CS-NYSm physically cross-linked film in both slightly basic (pH = 7.4) and acidic fluids (pH = 4.2), while the prolonged release of NYSm from chemically cross-linked formulations is less evident and appears only in a close to neutral environment. The combination of the known bioadhesion of chitosan with the strong antifungal effect manifested by nystatin was proved to be effective against yeasts of the genus *Candida* (*C. albicans*, *C. dubliniensis*, *C. glabrata*). Best results were obtained for the CS-NYSm physically cross-linked hydrogel, followed by the CS-NYSm-SA/DS-2 chemically cross-linked hydrogel (with smaller amount of DS). Nevertheless, the design of antifungal therapeutic systems remains challenging and of huge interest, this is the reason why we propose future research directions regarding the optimization of antifungal systems, the evaluation of the degradability of hydrogels (in vitro/in vivo) and their influence on drug release efficiency, as well as the testing of materials from a clinical point of view.

## 4. Materials and Methods

### 4.1. Materials

Chitosan (CS) with properties previously determined (degree of deacetylation of 81.6% and viscosimetric average molar mass of 290 kDa) [50], 1,3-bis(3-glycidyloxypropyl)tetramethyldisiloxane ≥ 95.0% (DS) and succinic anhydride (SA) were purchased from Merck Chemical (Saint Louis, MO, USA) and used as received. 

Anhydrous glycerin (Gly) and L-(+)-lactic acid (LA) were provided by Chemical Company (Iași, Romania), while micronized nystatin (NYSm) with 90% of particles with a size less than 10 µm (USP Reference Standard) was supplied by Antibiotice SA (Iași, Romania). *Candida albicans*-ATCC 90028, *Candida dubliniensis* ATCC MYA-178 and *Candida glabrata* ATCC 90030 were provided by American Type Culture Collection (Manassas, VA, USA).

### 4.2. Preparation of Chemically and/or Physically Modified Chitosan Antifungal Formulation

Each NYSm-loaded chitosan formulation (Table 1) was prepared by dissolving 0.3 g of the polysaccharide into 10 mL of 2% (*v*/*v*) lactic acid solution under continuous stirring at 40 °C (400 rpm for 24 h). Micronized nystatin (NYSm) was dispersed in glycerin (5%) by adapting a method previously described in the literature (grinding to homogenization using a pestle and a mortar) [27]. The so-prepared dispersion was added to the CS solution and magnetically stirred for 24 h at 40 °C to obtain the hydrogel formulation stabilized by non-covalent interactions (CS-NYSm). For the preparation of the chemically cross-linked hydrogel, CS in lactic acid solution was treated with SA and a DS cross-linking agent, so that the total percentage of succinylated and cross-linked amino groups of CS was 84% of the number of deacetylated units (8% or 25% for SA and 76% or 59% for DS). Thus, for the preparation of a hydrogel formulation with an amino group substitution of 8% and cross-linking of 76%, 15 mg of SA was introduced in the 10 mL CS-LA solution and stirred at 600 rpm for 6 h (40 °C). After homogenization, 244 mg of DS was added, and the mixture was kept under magnetic stirring in an oil bath at the same temperature for 24 h. The NYSm in the glycerin mixture was then added to the modified chitosan dispersion and the stirring continued for 24 h, to yield the CS-NYSm-SA/DS-1 hydrogel formulation. Similarly, another hydrogel (CS-NYSm-SA/DS-2) with a higher content of succinyl units and a lower degree of cross-linking (0.45 g of succinic anhydride and 1.90 g of DS) was prepared. 

In addition to NYSm-loaded chitosan hydrogels, a non-charged CS-LA sample was prepared for analytical purposes. All the prepared hydrogels were investigated as such, or as dried gels obtained after pouring the mixture into Petri dishes and drying by slow solvent evaporation at room temperature. The solvent casting drying method was chosen to ensure that the final product contains the same amount of NYSm as the one introduced. Thus, thin flexible films with an average thicknesses of around 50–70 μm (determined using a handheld micrometer with an accuracy of 1 µm, Dial Thickness Gauge 7301, Mitoyuto Corporation, Kangagawa, Japan) were obtained.

### 4.3. Methods of Characterization

The molecular docking computations were performed using the AutoDock-VINA algorithm [51], which is encompassed in the YASARA-Structure program package (v.20.8.23) [26].

Structural characterization of the dried gels was performed by Fourier transform infrared spectroscopy (FTIR) using a Bruker Vertex 70 spectrophotometer (Bruker Optics, Ettlingen, Germany). The spectra were recorded in ATR (Attenuated Total Reflectance) transmittance mode, in the wavelength range of 4000–600 cm^−1^, with a 2 cm^−1^ resolution and 64 scans at room temperature.

Scanning electron microscopy was used to morphologically investigate the surface and cross-section morphology of the CS films, using a FEI QUANTA 200 scanning microscope (Brno, Czech Republic) with a resolution of 4 nm at 30 kV. The energy-dispersive X-ray spectrometer (EDX) of the QUANTA 200 system was used to prove the chemical composition of the film formulations. Pore size distribution histograms were developed after processing SEM images from the chitosan films’ cross-section. Thus, the number and diameter of the pores were determined using the NIH Image J software, and the collected data were subsequently fitted with the Gaussian function (Gaussian multi peak) using OriginPro 8.5 software. 

Mechanical properties, such as elongation at break, tensile strength and Young’s modulus, were evaluated using a two-column Instron 3365 device, with a 500 N force cell (Norwood, MA, USA), on dumbbell-shaped cut samples with dimensions of 50 mm length, 8.5 mm width and 4 mm active width (ASTM D638 standard). Measurements were run at a strain rate of 50 mm/min at room temperature. For each sample, five specimens were analyzed and the average values were taken into consideration. According to Equations (1) and (2), both the strain (ε) and stress (σ) at break were determined:

ε (%) = Δl/l_0_ × 100, (1)

σ (MPa) = F/A,(2)

where l_0_ and ∆l represent the initial length of the sample and the elongation, respectively, F denotes the breaking force and A indicates the cross-sectional area at time t. Young’s modulus for each film was also calculated based on the specific deformation curve (as tensile stress/strain ratio at 1% deformation).

Swelling behavior of the NYSm-loaded CS films was evaluated in simulated physiological conditions, at two different pHs. Thus, the films were dried in an oven at 40 °C and immersed in phosphate-buffered saline (PBS) solution with a pH of 7.4 (simulating the saliva pH for oral administration) and in a solution of PBS with a pH adjusted to 4.2 by droplet addition of 1 M HCl (simulating the pH from a vaginal medium) [31]. The samples were maintained in the immersion medium at 37 °C and subjected to gentle shaking at 80 rpm (Orbital Shaker-Incubator ES-20/60, Biosan, Riga, Latvia), being extracted at predetermined time intervals for weighing (after PBS excess removal with filter paper). The swelling capacities of each modified CS film were gravimetrically determined using Equation (3):

S_t_ (%) = (w_t_ − w_0_)/w_0_ × 100, (3)

where S_t_ (%) gives the swelling capacity value at time t (min), w_0_ and w_t_ represent the initial mass (dried hydrogel) and the swollen hydrogel sample, respectively, at time t. All the experimental swelling kinetics data were processed using SCILAB 6.1.0 software by applying three mathematical models. Pseudo-first order (PFO) and pseudo-second order (PSO) models were applied to determine the swelling rate of the hydrogels in the PBS solution at equilibrium (Equations (4) and (5)) [45], while the Korsmeyer and Peppas (K–P) adapted model was used to evaluate the swelling dynamics of aqueous solution into the polymeric matrix (Equation (6)) [46,48]:

S_t1_ (%) = S_e1_^2^ (1 − exp (−k_s1_ × t), (4)

S_t2_ (%) = k_s2_ × S_e2_^2^/(1 + k_s2_ × S_e2_ × t), (5)

F = S_t3_/S_e3_ = k_p_ × t^n^, (6)

where S_t1_, S_t2_ and S_t3_ define the swelling capacities of the films at time t (min), S_e1_, S_e2_ and S_e3_ represent the swelling contents at equilibrium, k_1_ and k_2_ are constants for the swelling rate, while k_p_ is a constant which depends on the polymeric matrix, F represents the swelling fraction and n is the diffusion coefficient of the PBS solution into the dried hydrogels [45,46]. The diffusion parameter (n) provides information regarding the diffusion type mechanism of water molecules into the polymeric network as follows: 

n < 0.5 denotes a Fickian diffusion-controlled mechanism; n values between 0.5–1 indicate an anomalous non-Fickian diffusion, also n = 1 and n > 1 correspond to a relaxation-controlled water transport and supercase II diffusion, respectively [44,45,46].

In vitro micronized-nystatin release from film formulations was evaluated in both pH 7.4 and pH 4.2 buffer solutions. Thus, the dried hydrogels were immersed in the solutions and placed in the Orbital Shaker-Incubator (37 °C and 80 rpm). At different time intervals, a predetermined amount from each solution was extracted and analyzed using a UV-VIS spectrophotometer Hitachi U-3900 (Krefeld, Germania), afterwards being replaced with the same volume of initial PBS solution. Thus, the three characteristic absorption bands of the NYSm were identified (293 nm, 305 nm and 320 nm) as shown in Figure S7 and the calibration curves were plotted for the 320 nm adsorption band for both pH 7.4 and pH 4.2 (Figure S8a and Figure S8b, respectively). NYSm concentrations in PBS solutions were determined, and the release efficiency of the drug was assessed as a function of time. The experimental data were further processed by mathematically fitting with the PFO model, which describes a process dependent on concentrations (Equation (7)) [52] and the Fick’s Law/K–P semi-empirical equation (Equation (8)), in order to establish the type of NYSm release mechanism (Fickian or non-Fickian diffusion) and the stability behavior of the systems [53]:

S_r_ (%) = S_0_ (1 − exp (−k_r_ × t), (7)

M_t_/M_∞_ = k_pr_ · t^n^, (8)

where S_r_ and S_0_ represent the amount of drug released at time t and the initial amount of the drug in solution, k_r_ is the release constant, M_t_/M_∞_ is the fraction of drug release at a specific contact time t, M_t_ and M_∞_ refer to the drug released at time t and at infinite time, respectively, k_pr_ and n represent the gel characteristic constant and the diffusion coefficient, respectively. The type of drug release mechanism can be evaluated considering the *n* parameter value as follows: Fickian diffusion for n values smaller than 0.5, unidirectional or Fickian diffusion if n is equal to 0.5, anomalous or non-Fickian transport for n values higher than 0.5, Case II transport for n = 1 and supercase II transport for n > 1 [48].

The antifungal activity of NYSm chitosan hydrogels with nystatin was evaluated on three strains of Candida (*C. albicans*, C. *glabrata* and *C. dubliniensis*) using the agar disk diffusion test, according to the AFST-EUCAST EDef. 7.1 methods [54]. The tests were performed in a Remel RPMI-1640 medium buffered with MOPS and supplemented with 2% glucose, previously poured in Petri dishes. The microplates were prepared in advance and kept at a temperature of −20 °C until their use. For verification, all experiments were conducted in triplicate.

## Figures and Tables

**Table 1:** Chemical composition of chitosan-based hydrogels containing micronized nystatin (NYSm).

Hydrogel/Film ^1^ Code	CS (g)	NYSm (mg)	Glycerin (g)	SA ^2^ (mmol)	DS ^2^ (mmol)
CS-LA	0.3	-	-	-	-
CS-NYSm	0.3	15	4	-	-
CS-NYSm-SA/DS-1	0.3	15	4	0.15	0.53
CS-NYSm-SA/DS-2	0.3	15	4	0.45	0.38

^1^ The hydrogels were investigated as such or were dried by solvent evaporation at room temperature. ^2^ The molar contents of SA and DS were calculated in order to have only 84% of free NH_2_ groups of chitosan modified (succinylated and cross-linked).

**Table 2:** Mechanical properties (tensile strength at break—σ, elongation at break—ε, and Young’s modulus—Y) of the CS films.

Film Formulation	σ ^1^ (MPa)	ε ^1^ (%)	Y ^2^ (MPa)
CS-LA	101.63 ± 2.24	4.31 ± 0.13	43.23
CS-NYSm	2.89 ± 0.07	74.40 ± 1.52	0.12
CS-NYSm-SA/DS-1	1.60 ± 0.04	34.43 ± 0.99	0.29
CS-NYSm-SA/DS-2	1.04 ± 0.03	4.41 ± 0.16	0.41

^1^ Average values of five measurements for each film ± standard error (for n = 5). ^2^ Y (MPa) was calculated from the plotted strain-stress curves obtained with the average values of the five measurements.

## Data Availability

Not applicable.

## Supplementary Materials

The following supporting information can be downloaded at: https://www.mdpi.com/article/10.3390/gels8080495/s1, Figure S1: FTIR spectra of drug-free chitosan film (CS-LA), micronized nystatin (NYSm) and NYSm-loaded chitosan film (CS-NYSm); Figure S2: Micronized nystatin: (a) SEM images of micronized nystatin powder and (b) particle size distribution histogram for NYSm powder processed using NIH Image J software; Figure S3: Surface morphology of: (a) unmodified chitosan film (CS-LA); (b) micronized-nystatin-charged chitosan film (CS-NYSm); (c) DS cross-linked chitosan film (CS-NYSm-SA/DS-1); Figure S4: Chemical composition determined on the chitosan films’ surface by EDX for: (a) CS-LA, (b) CS-NYSm and (c) CS-NYSm-SA/DS-1 formulations; Figure S5: The characteristic stress-strain curves of the chitosan films: (a) CS-LA, (b) CS-NYSm, (c) CS-NYSm-SA/DS-1 and (d) CS-NYSm-SA/DS-2 films; Table S1: Parameters of the kinetic models (PFO—pseudo-first order, PSO—pseudo-second order and K–P—Korsmeyer–Peppas) and the swelling capacity values (%) of micronized-nystatin-loaded chitosan films after 5 h and 24 h, respectively; Table S2: Release efficiency (%) of NYSm from chitosan formulation (after 5 and 24 h) in immersion medium with pH 7.4 and 4.2, respectively, and the release kinetic models (PFO—pseudo-first order and K–P—Korsmeyer–Peppas) parameters; Figure S6: Antifungal effect against *Candida albicans*, *Candida dubliniensis* and *Candida glabrata* of: (a) CS-NYSm, (b) CS-NYSm-SA/DS-1 and (c) CS-NYSm-SA/DS-2 hydrogels; Figure S7: Identification of maximum absorbance wavelengths for micronized nystatin (concentration in solution of 20 mg/L); Figure S8: Graphical representation of calibration curves at a wavelength of 320 nm, in the concentration range 0–20 mg/L, for micronized nystatin (NYSm) at: (a) pH 7.4 and (b) pH 4.2.
