# Supplementary material for: Evaluation of Physically and/or Chemically Modified Chitosan Hydrogels for Proficient Release of Insoluble Nystatin in Simulated Fluids
Source: Gels. 2022 Aug 10;8(8):495. doi: 10.3390/gels8080495 (PMC9407202; doi:10.3390/gels8080495)
Supplement: Supplementary file 1 [file gels-08-00495-s001.zip › gels-1834406-supplementary.pdf]

gels

MDPI

Supplementary Materials

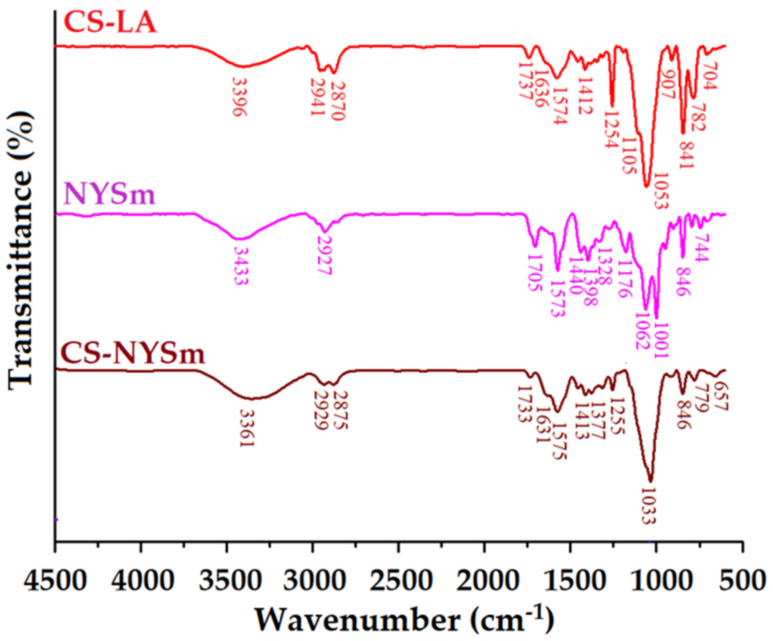

Figure S1. FTIR spectra of drug-free chitosan film (CS-LA), micronized nystatin (NYSm) and NYSm loaded chitosan film (CS-NYSm).

The CS-LA confirms the appearance of electrostatic interactions between protonated chitosan NH_3^+ groups and $^{-}$OOCCH(OH)CH_3 carboxylate ions (1737 cm^{-1}). Micronized nystatin shows specific absorption bands for C=O stretching vibrations (ester and carboxylic acid groups) and for the C=C polyenes units at 1705 cm^{-1}, respectively at 1573 cm^{-1} and 846 cm^{-1}. NYSm dispersion into the CS matrix determine the displacement of the C=O characteristic band from 1737 $^{-1}$ (CS-LA) to 1733 cm^{-1} (CS-NYSm), also the increase in intensity of band from 1575 cm^{-1} (C=C band of NYS superimposed with CS amide/amine bands) [25].

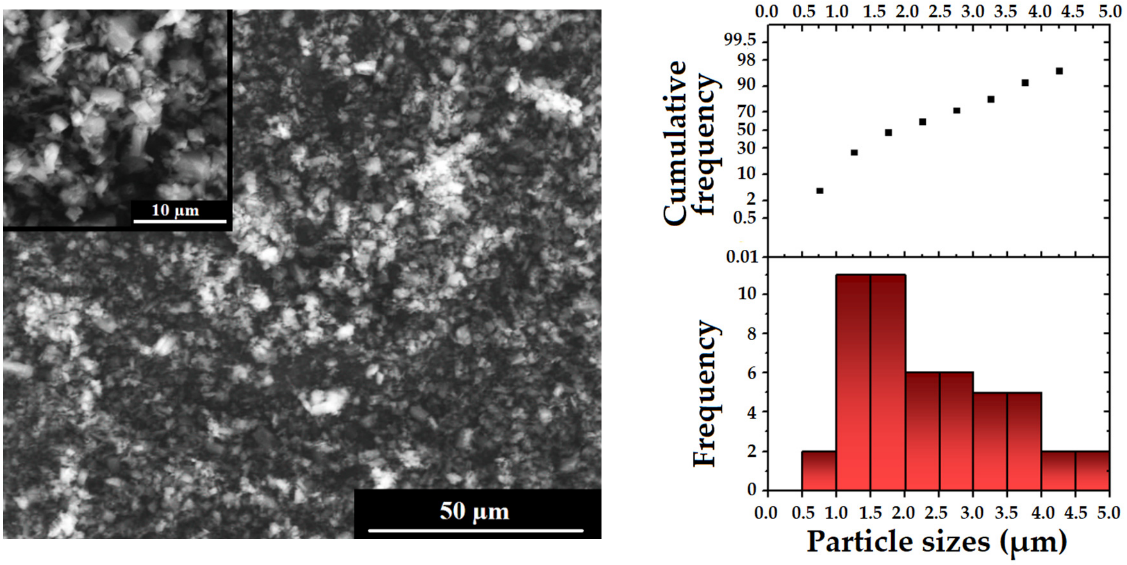

(**a**) (**b**)

Figure S2. Micronized nystatin: (a) SEM images of micronized nystatin powder and (b) particle size distribution histogram for NYSm powder processed using NIH Image J software.

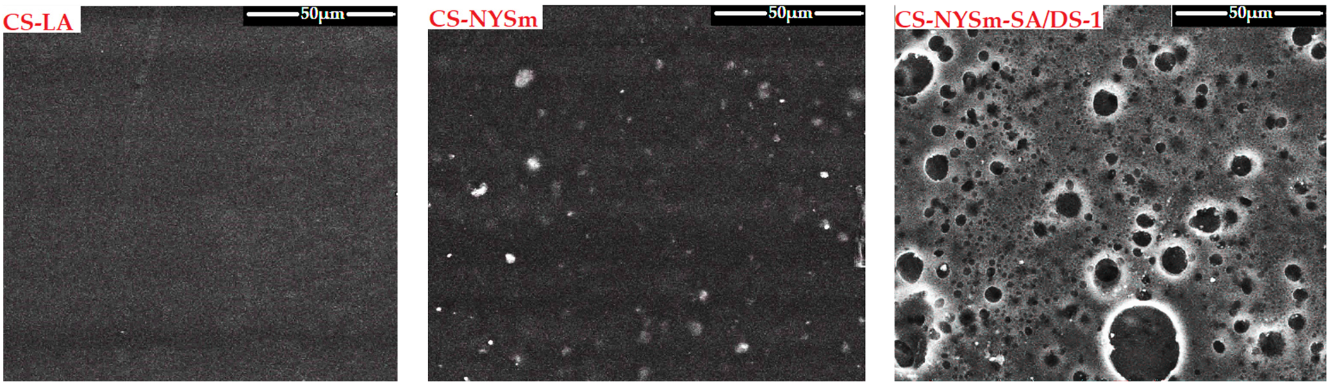

(**a**) (**b**) (**c**)

Figure S3. Surface morphology images of: (a) unmodified chitosan film (CS-LA); (b) micronized nystatin charged chitosan film (CS-NYSm); (c) DS cross-linked chitosan film (CS-NYSm-SA/DS-1).

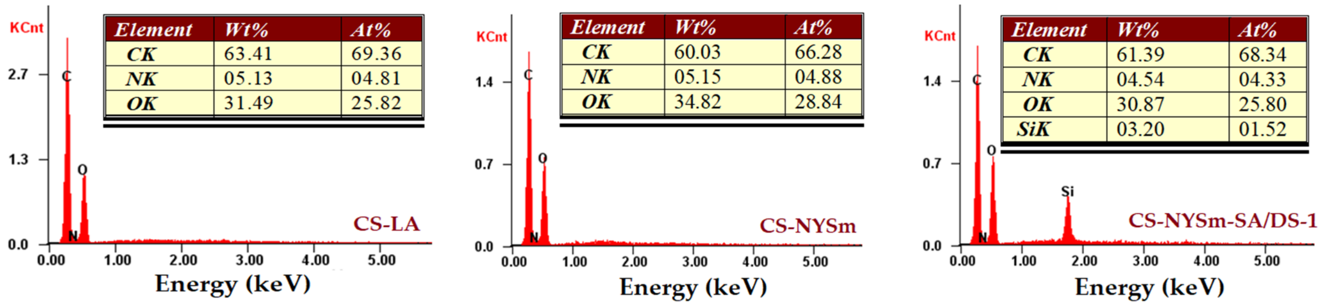

(**a**) (**b**) (**c**)

Figure S4. Chemical composition determined on the chitosan films surface by EDX for: (a) CS-LA, (b) CS-NYSm and (c) CS-NYSm-SA/DS-1 formulations.

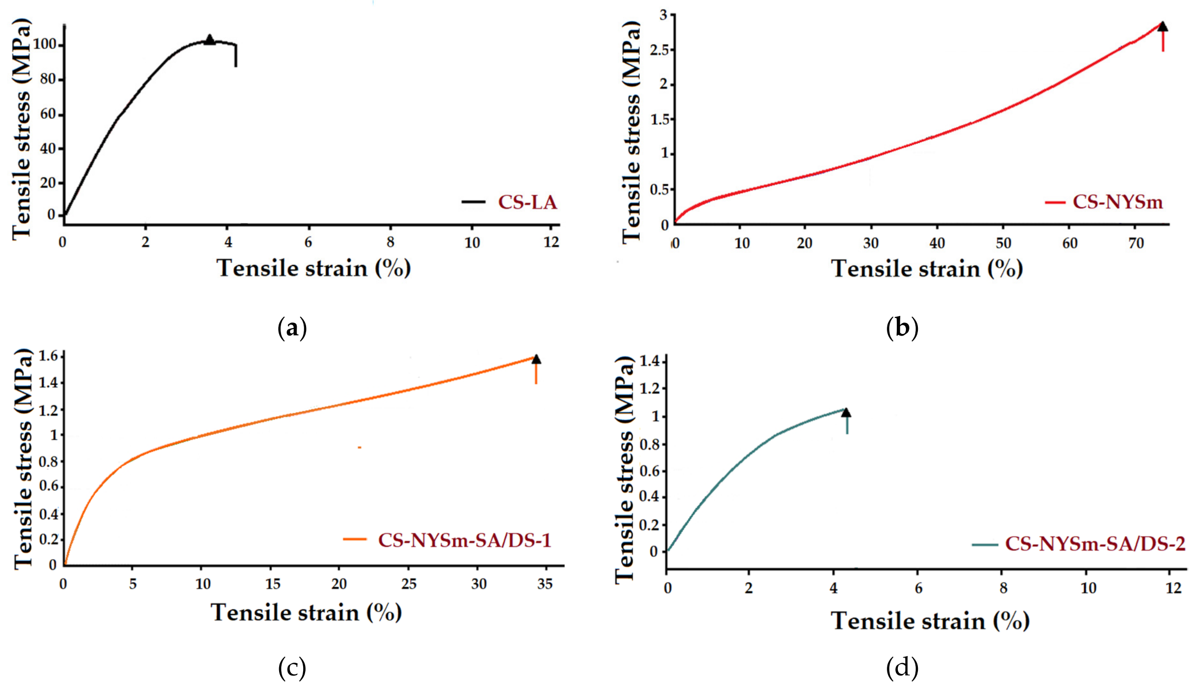

Figure S5. The characteristic stress-strain curves of the chitosan films: (a) CS-LA, (b) CS-NYSm, (c) CS-NYSm-SA/DS-1 and (d) CS-NYSm-SA/DS-2 films.

Table S1. Parameters [1] of the kinetic models (PFO - pseudo-first order, PSO - pseudo-second order and K-P - Korsmeyer-Peppas) and the swelling capacity values (%) of micronized nystatin loaded chitosan films after 5 h, respectively 24 h.

**Film code**	**pH**	**PFO model parameters**	**PSO model parameters**	**K-P model parameters**	**Swelling capacity after 5 h (%)**	**Swelling capacity after 24 h (%)**
**CS-NYSm**	7.4	$k_{s1} = 11.90$	$k_{s2} = 6.25 \cdot 10^{2}$	$k_p = 326.32$		
		$Se_1 = 338.84$	$Se_2 = 338.84$	$n = 1.04 \cdot 10^{-17}$	323	330
		SD = 3.56	SD = 3.42	SD = 5.33		
	4.2	$k_{s1} = 0.65$	$k_{s2} = 3.58 \cdot 10^{-3}$	$k_p = 285.86$	387	425
		$Se_1 = 356.59$	$Se_2 = 366.43$	$n = 0.05$		
		SD = 5.07	SD = 2.90	SD = 3.91		
**CS-NYSm-SA/DS-1**	7.4	$k_{s1} = 10.27$	$k_s = 1.88 \cdot 10^{3}$	$k_p = 232.40$		
		$Se_1 = 238.00$	$Se = 238.00$	$n = 9.97 \cdot 10^{-18}$	231	232
		SD = 1.81	SD = 1.81	SD = 2.54		
	4.2	$k_{s1} = 10.06$	$k_s = 1.90 \cdot 10^{2}$	$k_p = 210.45$	210	211
		$Se_1 = 211.20$	$Se = 211.20$	$n = 7.58 \cdot 10^{-18}$		
		SD = 0.89	SD = 0.89	SD = 0.92		
**CS-NYSm-SA/DS-2**	7.4	$k_{s1} = 10.83$	$k_s = 4.01 \cdot 10^{-3}$	$k_p = 335.80$		
		$Se_1 = 392.94$	$Se = 412.28$	$n = 4.26 \cdot 10^{-2}$	429	432
		SD = 9.94	SD = 3.94	SD = 4.89		
	4.2	$k_{s1} = 11.98$	$k_s = 2.74 \cdot 10^{-3}$	$k_p = 329.76$	471	500
		$Se_1 = 422.14$	$Se = 448.82$	$n = 0.06$		
		SD = 14.72	SD = 9.15	SD = 3.62		

[1] k_{s1} and k_{s2} are constants for the swelling rate, S_{e1} and S_{e2} represents the theoretical equilibrium swelling capacity, k_p represents a constant which is polymeric network dependent and n is the diffusion coefficient of aqueous buffer solution into the films formulations (SD = standard deviation for 11 data points).

Table S2. Release efficiency (%) of NYSm from chitosan formulation (after 5 and 24 h) in immersion medium with pH 7.4, respectively 4.2 and the release kinetic models (PFO - pseudo-first order and K-P - Korsmeyer-Peppas) parameters [1]

Film code	pH	PFO model parameters	K-P model parameters	NYSm release eficiency after 5 h (%)	NYSm release eficiency after 24 h (%)
**CS-NYSm**	7.4	k_r = 0.02<br>Sr = 46.85<br>SD = 0.96	k_{pr} = 7.44<br>n = 0.34<br>SD = 0.75	51	57
	4.2	k_r = 0.01<br>Sr = 57.35<br>SD = 0.68	k_{pr} = 5.45<br>n = 0.43<br>SD = 0.99	60	75
**CS-NYSm-SA/DS-1**	7.4	k_r = 0.05<br>Sr = 21.67<br>SD = 0.56	k_{pr} = 6.25<br>n = 0.24<br>SD = 0.47	23	24
	4.2	k_r = 0.04<br>Sr = 23.23<br>SD = 0.51	k_{pr} = 5.48<br>n = 0.27<br>SD = 0.52	24	33
**CS-NYSm-SA/DS-2**	7.4	k_r = 0.06<br>Sr = 31.88<br>SD = 0.63	k_{pr} = 9.39<br>n = 0.23<br>SD = 0.84	33	34
	4.2	k_r = 0.04<br>Sr = 23.95<br>SD = 0.50	k_{pr} = 5.78<br>n = 0.27<br>SD = 0.65	25	35

[1] k_r is the release constants and S_r represents the theoretical drug release, k_{pr} is a gel characteristic constant which and n is the diffusion coefficient of NYSm (SD = standard deviation for 11 data points).

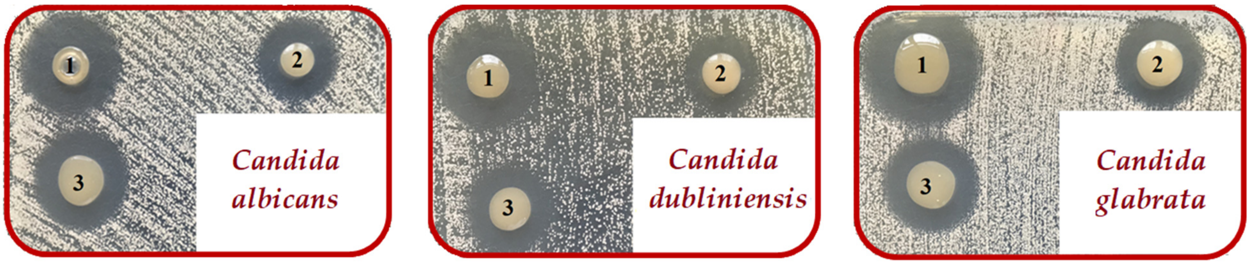

(a) (b) (c)

Figure S6. Antifungal effect against *Candida albicans*, *Candida dubliniensis* and *Candida glabrata* of: (a) CS-NYSm , (b) CS–NYSm-SA/DS-1 and (c) CS–NYSm-SA/DS-2 hydrogels.

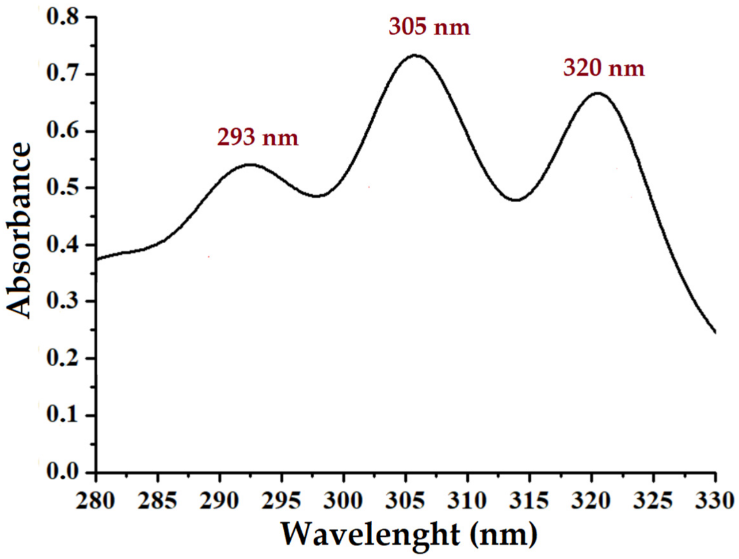

Figure S7. Identification of maximum absorbance wavelengths for micronized nystatin (concentration in solution of 20 mg/L).

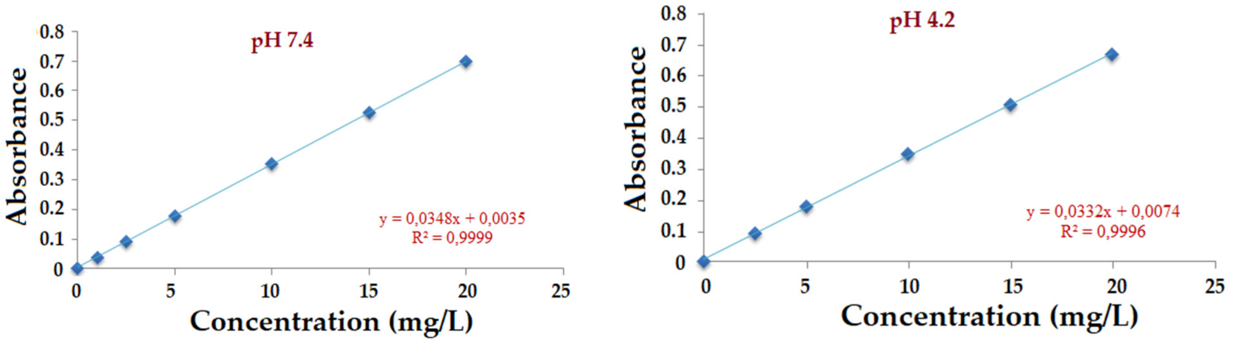

(**a**) (**b**)

Figure S8. Graphical representation of calibration curves at a wavelength of 320 nm, in the concentration range 0-20 mg/L, for micronized nystatin (NYSm) at: (a) pH 7.4 and (b) pH 4.2.
